# Synthesis and Properties of Chiral Thioureas Bearing an Additional Function at a Remote Position Tethered by a 1,5-Disubstituted Triazole

**DOI:** 10.3390/molecules15118327

**Published:** 2010-11-15

**Authors:** Kiyosei Takasu, Takumi Azuma, Iderbat Enkhtaivan, Yoshiji Takemoto

**Affiliations:** Graduate School of Pharmaceutical Sciences, Kyoto University, Yoshida, Sakyo-ku, Kyoto 606-8501, Japan; E-Mails: taku@f05azu.mbox.media.kyoto-u.ac.jp (T.A.), e.iderbat@ks8.ecs.kyoto-u.ac.jp (I.E.)

**Keywords:** multifunctional catalyst, thiourea, Ru-catalyzed Huisgen cycloaddition, asymmetric induction, hydrogen bonding

## Abstract

The synthesis and properties of multifunctional thioureas bearing a variety of functional groups at a position remote from the thiourea moiety are described. A 1,5-disubstituted triazole tether connected with a thiourea and another functional group was synthesized via ruthenium catalyzed Huisgen cycloaddition. We demonstrate the utility of the synthetic thioureas as asymmetric catalysts and probes for the mechanistic elucidation of the course of the Michael reaction of an α,β-unsaturated imide.

## 1. Introduction

**Figure 1:**
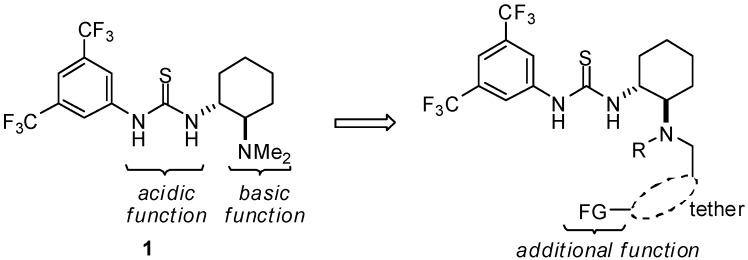
Design of a trifunctional thiourea catalyst.

The development of organocatalysts represents an important field in asymmetric synthesis [1]. Over the past decade, thiourea-based bifunctional catalysts such as **1** (Figure 1) have emerged as promising chiral catalysts due to ease of accessibility and their high efficiency in various asymmetric transformations [2,3,4,5,6,7,8,9]. Thiourea **1** has isolated acidic and basic functional groups in the same molecule. The combination of two functional groups within a chiral space of the catalyst leads to synergistic effects on the activation of substrates, providing high stereoselectivity and/or acceleration of the reaction rates. In most bifunctional thiourea catalysts, another functional partner is placed at a neighboring position so as to entropically activate the bimolecular reaction. Thioureas bearing another activating site at comparably remote positions have not been explored thoroughly [10]. On the other hand, in case of well-designed enzymes, sequentially distant functional groups can synergistically participate in the activation of the enzymatic reaction through the organization of an adequate chiral space. We envisioned that thiourea catalysts tethered with the third functional group at a remote position would provide further advantages with regard to molecular catalysis (Figure 1). One of the important and challenging matters would be the adequate design of the tether, which must appropriately display conformational flexibility/rigidity in order to provide an organized reaction space. Additionally, synthetic accessibility in the formation of the tether would be valuable.

**Figure 2:**
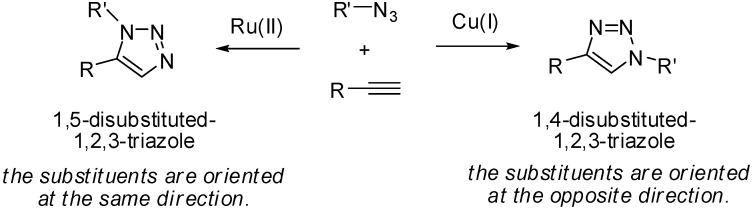
Regioisomeric 1,2,3-triazoles synthesized by Huisgen cycloaddition.

1,3-Dipolar cycloaddition of alkynes with alkyl- or arylazides, also known as the Huisgen cycloaddition [11], affords substituted 1,2,3-triazole compounds. A great deal of attention has been paid to the Cu(I)-catalyzed Huisgen cycloaddition giving 1,4-disubstituted triazoles (known as “click chemistry”) due to several synthetic advantages, including a wide tolerance for various functional groups, high chemical yield, simple reaction operation and easy purification [12]. Ru(II) catalysts are also known to activate the cycloaddition, but the catalyst results in the exclusive formation of 1,5-disubstituted triazoles [13,14,15]. Thus, both substituents of Ru-catalyzed cycloadducts, 1,5-triazoles, direct at the same side, whereas those of 1,4-disubstituted triazoles are oriented at opposite sides (Figure 2). We envisioned that the 1,5-disubstituted triazole core would be suitable for the tether for the following reasons: 1) conformational rigidity of the aromatic ring, 2) both substituents of 1,5-disubstituted triazoles directing to the same side, and 3) synthetic convenience. Herein we wish to report on the synthesis of chiral thioureas bearing acidic, basic or neutral functional groups at a remote position using Ru-catalyzed Huisgen cycloaddition and their utility as chiral organocatalysts and probes for the mechanistic elucidation of the course of the Michael reaction of an α,β-unsaturated imide [16].

## 2. Results and Discussion

### 2.1. Synthesis of Multifunctional Thioureas Bearing a 1,2,3-Triazole Tether

**Scheme 1:**
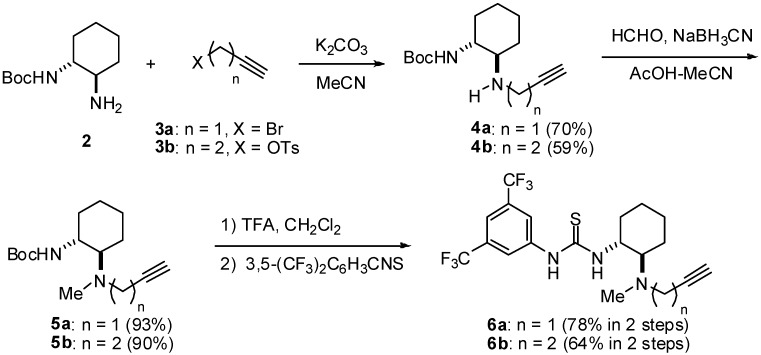
Synthesis of chiral bifunctional thioureas bearing an alkynyl moiety.

At the outset of this study, thioureas **6a,b** bearing an alkynyl moiety were synthesized (Scheme 1). Starting from mono-Boc 1,2-diaminocyclohexane (**2**) [17], alkylation with propargyl bromide (**3a**) and homopropargyl tosylate (**3b**) afforded secondary amines **4a** and **4b**, respectively. *N*-methylation of **4** was accomplished by reductive amination with formaldehyde to give **5**. Deprotection of the Boc group followed by treatment with 3,5-bis(trifluoromethyl)phenylisothiocyanate provided compounds **6** in good yield.

**Scheme 2:**
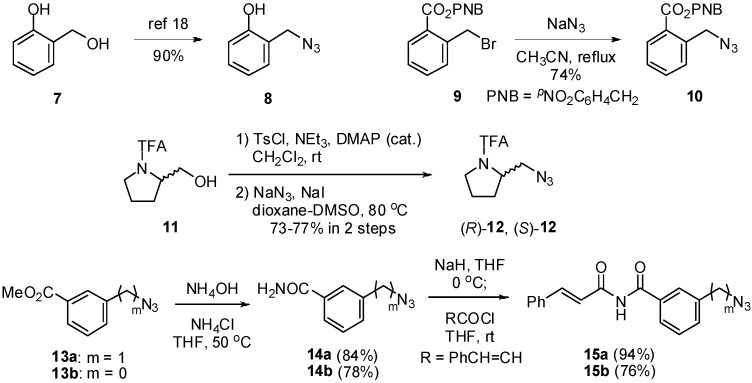
Synthesis of azide partners.

We envisioned synthesizing thioureas bearing a variety of functional groups at remote positions. Preparative procedures of azide partners **8**, **10**, **12**, **15** are depicted in Scheme 2. Azide **8** bearing a phenolic function was synthesized from alcohol **7** according to the reported procedure [18]. Azide **10** possessing a carboxylate equivalent was prepared from bromide **9** [19]. Both enantiomers of proline derivative **12** were obtained from the corresponding enantiomeric alcohols **11** [20] in two steps. Azides **15a,b** having α,β-unsaturated imide moieties were prepared in two steps from **13a,b** [21,22], respectively.

**Scheme 3:**
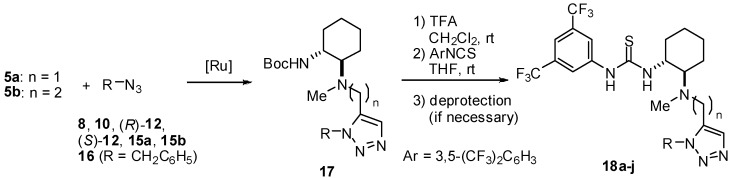
Synthesis of chiral thioureas bearing a 1,5-disubstituted triazole tether

We next examined the Ru-catalyzed Huisgen reaction of alkynes **6** with benzylazide (**16**) in the presence of Cp^*^Ru(PPh_3_)_2_Cl or [Cp^*^RuCl]_4_, which were reported to be highly active catalysts in the Huisgen cycloaddition [13,14,15]. Unfortunately, almost no formation of the desired cycloadduct was observed under any of the conditions tested. Further study revealed that the ruthenium catalyst was being inactivated by an undesired ligation with the sulfur atom of the thiourea moiety. Therefore, we modified the synthetic route towards creation of the desired thioureas to the following sequence: i) Ru-catalyzed Huisgen cycloaddition of alkynyl substrates and azide partners, ii) installation of thiourea moiety (Scheme 3). 

Chemical yields of **18** are summarized in Table 1. For example, regioselective Huisgen cycloaddition of alkyne **5a** with benzylazide (**16**) was smoothly activated by [Cp^*^RuCl]_4_ in THF at ambient temperature giving 1,5-disubstituted triazole **17a** in 71% yield (entry 1). 

**Table 1:** Chemical yields of **17** and **18.**

Entry	Substrates	Final products		Huisgen reaction		deprotection method*^b^*	overall %yield of 18 from 17
				method*^a^*	%yield of 17		
1	**5a, 16**	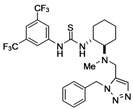	**18a**	**A**	71	-	51(2 steps)
2	**5a, 8**	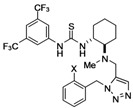	**18b **(X = OH)	**B**	42	-	57 (2 steps)
3	**5a, 10**		**18c **(X = CO_2_H)	**C**	71	**E**	41 (3 steps)
4	**5a**, (*R*)-**12**	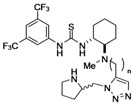	**18d** (n = 1, β-isomer)	**C**	54	**F**	71 (3 steps)
5	**5a**, (*S*)-**12**		**18e** (n = 1, α-isomer)	**C**	47	**F**	66 (3 steps)
6	**5b**, (*R*)-**12**		**18f** (n = 2, β-isomer)	**C**	43	**F**	58 (3 steps)
7	**5a, 15a**	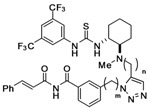	**18g** (n = 1, m = 1)	**A**	56	-	61 (2 steps)
8	**5a, 15b**		**18h** (n = 1, m = 0)	**A-D**	trace	-	-
9	**5b, 15a**		**18i** (n = 2, m = 1)	**D**	62	-	66 (2 steps)

*^a ^*Method **A**: Cp^*^Ru(PPh_3_)_2_Cl (10 mol%), THF, rt, 24 h; method **B**: [Cp^*^RuCl]_4_ (2.5 mol%), DMF, 110 °C, microwave, 20 min; method **C**: [Cp^*^RuCl]_4_ (2.5 mol%), THF, reflux, 8 h; method **D**: [Cp^*^RuCl]_4_ (2.5 mol%), DMF, 110 °C, microwave, 1 h. *^b^*Method **E**: LiOH (10 eq), THF-H_2_O, rt, 10 h; method **F**: LiOH (10 eq), THF-H_2_O, rt, 2-4 h.

Conversely, the reaction with phenol **8** under the same conditions resulted in poor conversion to the desired triazole **17b**. It was found that microwave irradiation in DMF (110 ^o^C) was effective for the cycloaddition, giving **17b** in 42% yield (entry 2). Huisgen cycloaddition of **5a,b** with azides **8**, **10**, **12, 15a** and **16** , respectively, under the same conditions, afforded **17** in moderate yield (entries 2-7 and 9). The regioselectivity in the cyloaddition was controlled to furnish 1,5-disubstited 1,2,3-triazoles exclusively. However, it was found that the reactivity of arylazide **15b** in the Ru-catalyzed Huisgen reaction was poor and, consequently, only a trace amount of triazole **17h** was produced under all conditions tested (entry 8). Transformation of **17** into **18** was achieved over a couple of steps, namely, deprotection of the Boc group of **17**, followed by treatment with 3,5-bis(trifluoromethyl)isothiocyanate and hydrolysis (only for **17c-f**), furnished thiourea **18a-g,i** in fair yield. The synthetic sequence involving Huisgen cycloaddition would be a facile and new methodology to prepare new classes of multifunctional thioureas. Although a thiourea function is incompatible, it was found that various functionalities, such as phenol, amine, amide, carbamate, imide and ester groups, are tolerant of the Ru-catalyzed Huisgen reaction.

**Scheme 4:**
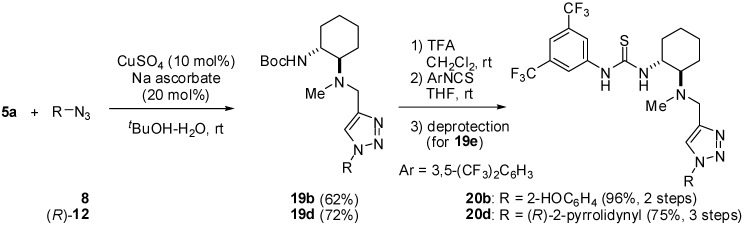
Synthesis of chiral thioureas bearing a 1,4-disubstituted triazole tether.

Thiourea catalysts having a regioisomeric 1,4-disubstituted triazole tether were also synthesized using Cu(I)-catalyzed cycloaddition (Scheme 4). 1,3-Dipolar cycloaddition of alkyne **5a** with **8** and (*R*)-**12** in the presence of a catalytic amount of Cu(II) salt with a reductant [23] furnished 1,4-disubstituted 1,2,3-triazoles **19b** and **19d**, respectively. According to the same method as above, thioureas **20b** and **20d** were obtained in good overall yield.

### 2.2. Michael Reaction of α,β-Unsaturated Imides Bearing a Thiourea Auxiliary: Identification of Adequate Hydrogen Bond Network

**Figure 3:**
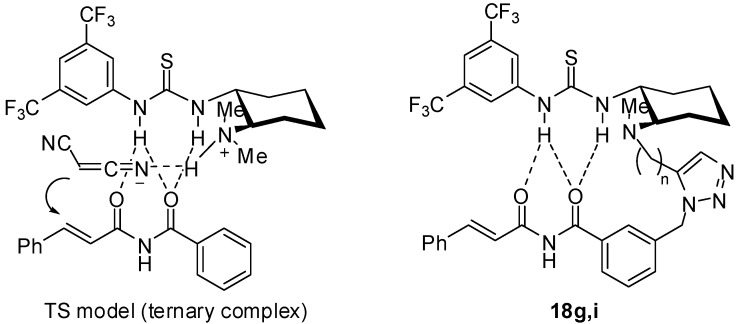
A transition state model for a Michael addition to imide with bifunctional thiourea **1** (left), and its mimetic hybrid molecules **18g,i** (right).

We have reported that thiourea **1** smoothly catalyzes a conjugate addition of malononitrile to α,β-unsaturated imides to give the corresponding Michael adducts with high enantioselectivity [24,25,26]. We proposed a ternary complex as the transition state model [27], in which the thiourea moiety of **1** would interact with the imide function of the substrate by two sets of hydrogen bonding to create an adequate chiral catalytic site, and, moreover, malononitrile would be activated by an amino moiety of **1** (Figure 3, *left*). However, because the binding constant of thiourea **1** with an imide substrate was very small, it was difficult to observe the binding structure by NMR in order to elucidate the mechanistic insight. The correct structure of the transition state remains to be cleared. We envisaged that thioureas **18g** and **18i** tethered with an α,β-unsaturated imide moiety would be utilized as appropriate mimic for the transition state model.

**Scheme 5:**
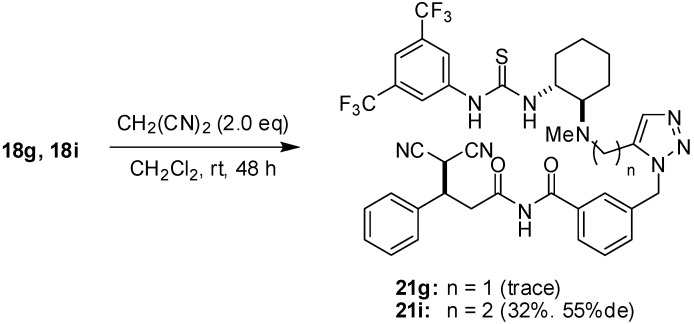
Michael addition of **18g** and **18i** with malononitrile.

For this purpose, we examined the reactivity of **18g** and **18i** in a Michael addition with malononitrile. If the thiourea moiety of **18** interacts with the imide group via the appropriate hydrogen bonds like the transition state shown in Figure 3, the conjugate addition should proceed much more smoothly as compared with a substrate possessing no or less hydrogen bond Interaction. When **18g** was treated with two equivalents of malononitrile in dichloromethane at room temperature, almost no reaction producing Michael adduct **21g** occurred within 48 h (Scheme 5). In contrast, **18i**, whose tether is one methylene unit longer than that of **18g**, furnished the corresponding adduct **21i** in 32% yield [28]. The diastereomeric selectivity of **21i** was determined to be 55%de after transesterification of **21i** to give the corresponding methyl ester. The chirality of the stereogenic center was assigned to be (*R*), which is identical to that reported ones using thiourea **1** [24]. The results indicated that the α,β-unsaturated imide moiety would be activated by the thiourea function through the hydrogen bonding and the length of the tether between thiourea and imide functions would be very important. Although we next attempted the conformational analysis of **18g,i** to throw light on their hydrogen bond network, no suitable crystal on which X-ray crystallography could be performed was obtained and it was, unfortunately, difficult to analyze the conformation via NMR. We anticipate that further studies employing another approach will be indispensable to elucidate the transition state for the Michael addition catalyzed by thiourea **1.**

### 2.3. Asymmetric Michael addition with Thiourea-Pyrrolidine Based Trifunctional Catalyst

We next examined the utility of trifunctional catalysts **18** and **20 **having a triazole tether to elucidate the effect of the remote functional group. Asymmetric Michael addition is one of the representative C‑C bond formation reactions in organocatalysis. In particular, extensive efforts have been devoted to the enantioselective Michael reaction of ketones with nitroalkenes [29,30,31,32,33,34,35,36,37,38] since the nitroalkanes produced bearing contiguous stereogenic centers would be versatile synthetic intermediates. Several pyrrolidine-based derivatives have been reported to catalyze the reaction with good to high diastereo- and enantioselectivity. Chiral thiourea-pyrrolidine-based bifunctional catalysts have been also found to give excellent enantioselectivities [7]. However, some problems, such as the slow reaction rates still persist with most of the pyrrolidine-based organocatalysts. 

During the course of our study, Kilburn *et al.* reported on some thiourea-pyrrolidine based bifunctional catalysts [7] in which both functions are placed at considerably distant positions tethered with a simple alkyl chain. Some of these bifunctional catalysts demonstrated excellent rate acceleration with good stereoselectivity in the reaction of cyclohexanone with *trans*-β-nitrostyrene. They clarified the fact that the tether between thiourea and pyrrolidine of the optimized catalyst consists of five atoms.

The catalytic activity of thiourea-pyrrolidine catalyst **18d-f** and **20d** was evaluated under the same conditions as Kilburn’s study [7] (Table 2). The thiourea moiety of **18d** and **18e** is separated from the imide function by seven atoms, whereas the spacing of **18f** and **20d** is eight atoms. Catalyst **18d** which has a 1,5-disubstituted triazole tether produced nitroalkane **23a** in 91% yield with good diastereo- and enantioselectivities (91:9 *syn*/*anti* selectivity, 92% ee of *syn*-**23a**; entry 1). The stereochemistry of major isomer **23a** was determined to be *syn* by comparison with reported data [5,6,7,8,9]. The chirality of **23a** obtained from **18d** was opposite to that from **18e** (entry 2). Thus, the enantioselection in the reaction appears to be mainly dominated by the chirality of the pyrrolidinyl moiety. Although the difference in the value of enantiomeric excess is not so significant, it was observed that the chirality of the 1,2-diaminocyclohexyl moiety affects the selectivity somewhat (entries 1 vs 2). Catalyst **18f** having a tether that is one methylene longer also afforded **23a** in good yield, however, with lower *syn/anti* selectivity and enantioselectivity (entry 3). The results clearly indicated that tether length would be important for asymmetric induction in the Michael addition. Interestingly, we have found that the rate of reaction with **20d** having a 1,4-disubstituted triazole tether was much slower than that with **18d-f**, although the enantioselectivity was comparable to that of **18d** (entry 4). 

**Table 2:** Enantioselective conjugate addition of cyclohexanone to trans-β-nitrostyrene catalyzed by trifunctional thiourea. *^a^* 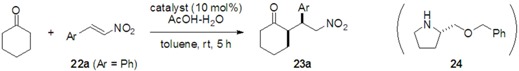

entry	catalyst	%yield of 23a*^b^*	dr (*syn*/*anti*)*^c^*	%ee of *syn*-23a*^d^*
1	**18d**	91	91 : 9	92
2	**18e**	93	91 : 9	82 (*ent*)
3	**18f**	85	82 : 18	55
4	**20d**	32	93 : 7	87
5*^e^*	**24**	10*^f^*	91 : 9	93 (*ent*)

*^a ^*The reaction was conducted with **22a** (0.34 mmol) and cyclohexanone (3.4 mmol, 10 equiv.) in the presence of catalyst (10 mol%), AcOH (15 mol%) and H_2_O (1.0 equiv) in toluene (0.5 mL) at ambient temperature. *^b ^*Isolated yield as a mixture of *syn*/*anti* isomers. *^c ^*Determined by HPLC analysis and ^1^H-NMR. *^d ^*Determined by HPLC analysis (Daicel Chiralpak AS-H, hexane-*^i^*PrOH = 90:10). *^e ^*The reaction result was cited from Kilburn’s study (ref. [7]). *^f ^*Conversion yield after the reaction was carried out for 720 h.

This result points out that the relative position of the thiourea and pyrrolidine moieties are a critical factor for the rate acceleration in the Michael addition reaction. Although the catalysts **18f** and **20d** possess a tether that is eight atoms in length, the acceleration rate of the conjugate addition by catalyst **18f** was, interestingly, greater than that of **20d**. Thus, the direction of the substituents on the triazole ring of the catalyst would affect the rate enhancement in the reaction. In other word, the tether of **18f** would be more flexible than that of **20d**. Therefore, both of the thiourea and pyrrolidine moieties of **18f** could participate in the synergistic activation of the substrates. 

**Figure 4:**
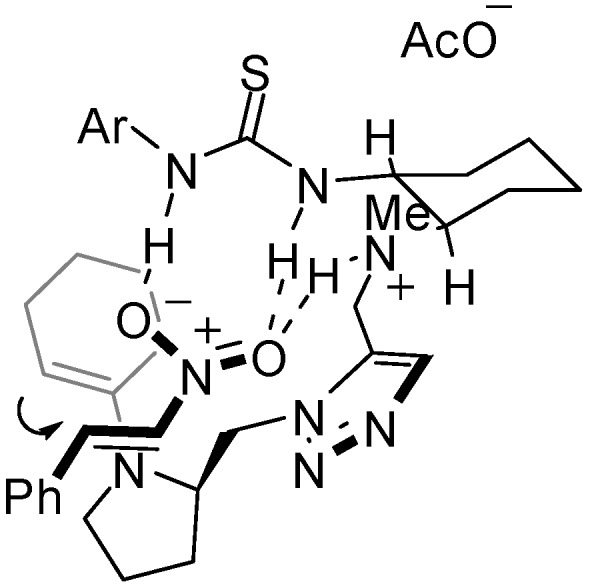
A Proposed Transition State for Michael Addition by Trifunctional Thiourea **18d**

As Kilburn reported that the reaction rate drastically decreased in the reaction with monofunctional pyrrolidine catalyst **24** (entry 5), it has been made clear that the thiourea function of the catalyst system can positively participate in the activation of the substrate. The absolute configuration of the major enantiomer *syn*-**23a** in the reaction with **18d** was determined to be (2*R*,1’*S*) by the comparison of HPLC data with reported data [5,6,7,8,9]. The configuration is consistent with a synclinal transition state for pyrrolidine-based chiral organocatalysis. A suggested transition state model is shown in Figure 4. The hydrogen bond network among the thiourea moiety, tertiary ammonium and the nitro group would direct the nitrostyrene to attack of *si*-face of the enamine.

Furthermore, we examined the scope of the asymmetric Michael addition using **18d** (Table 3). Nitroolefins **22b-f** bearing a variety of aryl group gave the corresponding Michael adducts in high yield with a good stereoselectivity [39].

**Table 3:** Scope of the Enantioselective Michael addition by catalyst **18d**.*^a^*

Entry	Substrate 22 (Ar)	% Yield of 5 *^b^*	dr (*syn*/*anti*)*^c^*	% ee of *syn*-23*^d^*
1	**22b** (2-ClC_6_H_4_)	88	91 : 9	91
2	**22c** (3-ClC_6_H_4_)	89	90 : 10	91
3	**22d** (4-ClC_6_H_4_)	83	89 : 11	89
4*^e^*	**22e** (4-MeOC_6_H_4_)	89	91 : 9	91
5	**22f** (2-furyl)	96	84 : 16	98

*^a ^*The reaction was conducted with **22** (0.34 mmol) and cyclohexanone (3.4 mmol, 10 equiv.) in the presence of **18d** (10 mol%), AcOH (15 mol%) and H_2_O (1.0 equiv) in toluene (0.5 mL) at ambient temperature. Reactions were carried out for 5 h (except for entry 4) *^b ^*Isolated yield as a mixture of *syn*/*anti* isomers. *^c ^*Determined by HPLC analysis and ^1^H-NMR. *^d ^*Determined by HPLC analysis (for **23b**: Daicel Chiralpak AD-H, hexane-*^i^*PrOH = 90:10; for **23c**: Chiralpak AS-H, hexane-*^i^*PrOH = 75 : 25; for **23d-h**: Chiralpak AD-H, hexane-*^i^*PrOH = 91:9). *^e^*The reaction was carried out for 12 h.

## 3. Experimental

### 3.1. General

All reactions were carried out under a positive atmosphere of argon in dried glassware unless otherwise noted. Solvents and materials were obtained from commercial suppliers and used without further purification. Column chromatography was performed on Merck silica gel 60 (230-400 mesh). Reactions and chromatography fractions were analyzed employing pre-coated silica gel plate (Merck Silica Gel 60 F_254_). All melting points were measured on YANACO MP-500P micro melting point apparatus and are uncorrected. IR spectra were measured on JASCO FT/IR-410. The ^1^H- and ^13^C- NMR spectra were recorded on JEOL AL-400 or JEOL ECP-500 instruments with tetramethylsilane as internal standard. Low-resolution and high-resolution mass spectra were recorded on JEOL JMS-01SG-2 or JMS-HX/HX 110A mass spectrometer. 

### 3.2. Representative Synthetic Procedure: Preparation of ***6a***

*tert-Butyl (1R,2R)-2-(2-Propynylamino)cyclohexylcarbamate* (**4a**): To a stirred mixture of **2 **(1.50 g, 7.0 mmol) and K_2_CO_3_ (1.03 g, 8.4 mmol) in MeCN (30 mL) at room temperature, propagyl bromide (832 mg, 7.0 mmol) in MeCN (40 mL) was added. After being stirred at room temperature for 3 h, the mixture was quenched with water (20 mL) and extracted with CHCl_3_ (100 mL × 3). The extracts were dried over NaSO_4, _filtered, and concentrated *in vacuo*. The residue was purified by silica gel column chromatography with hexane/AcOEt (1:1) to afford **4a** (1.24 g, 70%). Colorless crystals; [α]_D_^24^ -18.3 (*c* 0.94, CHCl_3_); Mp 109-110 °C; ^1^H-NMR (400 MHz, CDCl_3_) δ 4.46 (br, 1H), 3.52 (dd, *J* = 17.6, 2.4 Hz, 1H), 3.39 (dd, *J* = 17.6, 2.4 Hz, 1H), 3.32 (br, 1H), 2.45 (ddd, *J* = 10.4, 10.4, 6.0 Hz, 1H), 2.20 (dd, *J* = 2.4, 2.4 Hz, 1H), 2.04-2.06 (m, 1H), 2.05 (br, 1H), 1.66-1.73 (m, 1H), 1.45 (s, 9H), 1.04-1.42 (m, 4H) ppm; ^13^C-NMR (126 MHz, CDCl_3_) δ 155.9, 82.6, 79.4, 71.0, 59.3, 54.4, 35.3, 32.9, 31.1, 28.4, 24.8, 24.3 ppm; IR (ATR) 3349, 3313, 3251, 2973, 2935, 2859, 1718, 1679, 1519 cm^-1^; MS (FAB) 253 (MH^+^, 100); Anal. Calcd. for C_14_H_24_N_2_O_2_: C, 66.63; H, 9.59; N, 11.10; Found; C, 66.66; H, 9.73; N, 10.94.

*tert-Butyl (1R,2R)-2-{Methyl-(2-propynyl)amino}cyclohexylcarbamate* (**5a**): To a stirred mixture of **2a** (1.10 g, 4.4 mmol) in MeCN (30 mL) at room temperature, 37% *aq* HCHO (707 mg, 8.7 mmol) was added. After the mixture was stirred at room temperature for 15 min and 45 min, NaBH_3_CN (274 mg, 4.4 mmol) and AcOH (9 mL), respectively, were added. After being stirred at the same temperature for 4 h, the mixture was quenched with 1N *aq* NaOH (150 mL) and extracted with CHCl_3_ (150 mL × 3). The extracts were dried over NaSO_4, _filtered, and concentrated *in vacuo*. The residue was purified by silica gel column chromatography with hexane/AcOEt (8:1) to afford **5a** (1.08 g, 93%). Colorless oil; [α]_D_^24^ -41.9 (*c* 1.1, CHCl_3_);^ 1^H-NMR (400 MHz, CDCl_3_) δ 5.09 (br, 1H), 3.35 (t, *J* = 2.8 Hz, 2H), 3.21-3.30 (m, 1H), 2.40-2.46 (m, 2H), 2.28 (s, 3H), 2.20 (t, *J* = 2.8 Hz, 1H), 1.89-1.92 (m, 1H), 1.75-1.78 (m, 1H), 1.63-1.66 (m, 1H), 1.45 (s, 9H), 1.05-1.29 (m, 4H) ppm; ^13^C-NMR (126 MHz, CDCl_3_) δ 156.2, 81.4, 78.9, 72.2, 65.1, 51.9, 42.7, 36.1, 33.2, 28.5, 25.3, 24.5, 23.31 ppm; IR (ATR) 3311, 1694, 1484 cm^-1^; MS (FAB) 267 (MH^+^, 84), 211 (100); Anal. Calcd. for C_15_H_26_N_2_O_2_: C, 67.63; H, 9.84; N, 10,52; Found; C, 67.40; H, 10.11; N, 10.44.

*1-{3,5-Bis(trifluoromethyl)phenyl}-3-(1R,2R)-2-[{methyl(2-propynyl)amino}cyclohexyl]thiourea* (**6a**): To a stirred mixture of **5a **(100 mg, 0.38 mmol) in CH_2_Cl_2_ (1 mL) at room temperature, TFA (1 mL) was added. After being stirred at the same temperature for 3 h, the mixture was basified with 3 N NaOH*aq* (5 mL) and extracted with CHCl_3_ (5 mL × 3). The extracts were dried over NaSO_4, _filtered, and concentrated *in vacuo*. A mixture of the resulting crude product and 3,5-bis(trifluoromethyl)-phenylisothiocyanate (82 mg, 0.30 mmol) in THF (1.5 mL) was stirred at room temperature for 11 h. After concentration *in vacuo*, the mixture was purified by silica gel column chromatography with hexane/AcOEt/NEt_3_ (150:50:1) to afford **6a** (112 mg, 78% in two steps). Pale yellow oil; [α]_D_^24^ -17.5 (c 1.2, CHCl_3_);^ 1^H-NMR (400 MHz, acetone-*d*_6_) δ 9.47 (br, 1H), δ 8.28 (s, 2H), 7.67 (s, 1H), 7.51 (br, 1H), 4.25 (br, 1H), 3.44 (dd, *J* = 16.8, 2.4 Hz, 1H), 3.37 (dd, *J* = 16.8, 2.4 Hz, 1H), 2.77-2.84 (m, 1H), 2.64 (t, *J* = 2.4 Hz, 1H), 2.45-2.48 (m, 1H), 2.37 (s, 3H), 2.04-2.06 (m, 1H), 1.78-1.82 (m, 1H), 1.67-1.70 (m, 1H), 1.18-1.43 (m, 4H) ppm;^ 13^C-NMR (126 MHz, acetone-*d*_6_): 181.1, 142.7, 132.1 (q, *J_C-F_* = 33.7 Hz), 124.3 (q, *J_C-F_* = 273 Hz), 123.0, 117.2, 82.0, 73.8, 66.0, 56.4, 43.0, 36.5, 32.9, 25.9, 25.4, 24.2; IR (ATR) 3309, 2937, 1531, 1467 cm^-1^; MS (FAB) 438 (MH^+^, 100); HRMS (FAB^+^) [C_19_H_22_F_6_N_3_S]^+^: 438.1439; Found. 438.1432

*tert-Butyl (1R,2R)-2-(3-Butynylamino)cyclohexylcarbamate* (**4b**): Colorless crystals; [α]_D_^24^ -30.5 (c 1.0, CHCl_3_); Mp 89-90 °C; ^1^H-NMR (400 MHz, CDCl_3_) δ 4.60 (br, 1H), 3.22 (br, 1H), 2.84-2.90 (m, 2H), 2.66-2.72 (m, 1H), 2.33-2.40 (m, 2H), 2.23-2.30 (m, 1H), 2.09 (d, *J* = 12.0 Hz, 1H), 1.98-2.03 (m, 1H), 1.98 (dd, *J* = 2.4, 2.4 Hz, 1H), 1.65-1.72 (m, 2H), 1.45 (s, 9H), 1.12-1.32 (s, 4H) ppm; ^13^C- NMR (126 MHz, CDCl_3_) δ 155.9, 82.6, 79.2, 69.5, 60.5, 54.4, 44.7, 32.8, 31.6, 28.4, 24.8, 24.6, 19.9 ppm; IR (ATR) 3280, 2933, 2857, 1708, 1525 cm^-1^; MS (FAB) 267 (MH^+^, 100); Anal. Calcd. for C_15_H_26_N_2_O_2_: C, 67.63; H, 9.84; N, 10,52; Found; C, 67.36; H, 9.74; N, 10.35.

*tert-Butyl (1R,2R)-2-{3-Butynyl(methyl)amino}cyclohexylcarbamate* (**5b**): Colorless crystals; Mp 60-61 °C; [α]_D_^26^ -37.7 (*c* 1.6, CHCl_3_); ^1^H-NMR (400 MHz, CDCl_3_) δ 5.45 (br, 1H), 3.15-3.23 (m, 1H), 2.65-2.70 (m, 1H), 2.47-2.54 (m, 2H), 2.29-2.33 (m, 3H), 2.21 (s, 3H), 1.98 (t, *J* = 2.7 Hz, 1H), 1.59-1.78 (m, 3H), 1.44 (s, 9H), 1.03-1.26 (m, 4H) ppm; ^13^C-NMR (126 MHz, CDCl_3_) δ 156.4, 83.0, 78.6, 69.1, 66.4, 52.0, 36.1, 33.1, 28.5, 25.5, 24.5, 23.0, 18.6 ppm; IR (ATR) 3383, 2974, 2929, 2857, 2361, 1707, 1483 cm^-1^: MS (FAB) 281 (MH^+^, 100); Anal. Calcd. for C_16_H_28_N_2_O_2_: C, 68.53; H, 10.06; N, 9.99; Found; C, 68.38; H, 10.34; N, 9.78.

*1-{3,5-Bis(trifluoromethyl)phenyl}-(1R,2R)-3-[2-{2-butynyl(methyl)amino}cyclohexyl]thiourea* (**6b**): White amorphous; [α]_D_^27^ -20.2 (*c* 1.0, CHCl_3_); ^1^H-NMR (400 MHz, acetone-*d*_6_) δ 9.34 (br, 1H), δ 8.28 (s, 2H), 7.66 (s 1H), 7.47 (br, 1H), 4.18 (br, 1H), 2.78 (m, 1H), 2.64 (m, 1H), 2.48-2.55 (m, 2H), 2.23-2.32 (m, 2H), 2.30 (s, 3H), 2.03 (t, *J* = 2.4 Hz, 1H), 1.90-1.94 (m, 1H), 1.77-1.80 (m, 1H), 1.63-1.67 (m, 1H), 1.13-1.32 (m, 4H) ppm; ^13^C-NMR (126 MHz, acetone-*d*_6_) δ 181.3, 142.8, 132.1 (q, *J_C-F_* = 27.7 Hz), 126.5 (q, *J_C-F_* = 270 Hz), 123.1, 117.2, 83.6, 70.4, 66.9, 56.4, 52.6, 37.5, 33.0, 25.4, 23.9, 19.2 ppm; IR (ATR) 3195, 3047, 2935, 1530, 1467 cm^-1^; MS (FAB) 452 (MH^+^, 100); Anal. Calcd. for C_20_H_23_F_6_N_3_S: C, 53.27; H, 5.13; N, 9.37; Found; C, 53.25; H, 5.15; N, 9.31.

### 3.3. Synthesis of Azides

4-Nitrobenzyl 2-(Azidomethyl)benzoate (**10**): To a stirred solution of NaN_3_ (294 mg, 4.5 mmol) in MeCN (2 mL), **9 **(660 mg, 1.9 mmol) in MeCN (3 mL) was added at room temperature. The mixture was refluxed for 20 h. After water (10 mL) was added, the organic layer was extracted with AcOEt (10 mL × 3). The extracts were dried over MgSO_4, _filtered, and concentrated *in vacuo*. The resulting residue was purified by silica gel column chromatography with hexane/AcOEt (7:1) to afford **10** (433 mg, 74%). Colorless crystals; Mp 33-34 °C; ^1^H-NMR (400 MHz, CDCl_3_) δ 8.26 (d, *J* = 6.8 Hz, 2H), 8.08 (dd, *J* = 7.8, 1.2 Hz, 1H), 7.58-7.63 (m, 3H), 7.51 (d, *J* = 7.1 Hz, 1H), 7.42-7.46 (m, 1H), 5.46 (s, 2H), 4.82 (s, 2H) ppm; ^13^C-NMR (126 MHz, CDCl_3_) δ 166.0, 147.8, 143.0, 137.7, 133,2, 131,2, 130.1, 129.0, 128.3, 128.0, 123.9, 65.4, 53.1 ppm; IR (ATR) 2941, 2094, 1715, 1603 cm^-1^; MS (FAB) 313 (MH^+^, 13),136 (100); Anal. Calcd. for C_15_H_12_N_4_O_4_: C, 57.69; H, 3.87; N, 17.94; Found: C, 57.72; H, 3.64; N, 18.19.

*(S)-1-{2-(Azidomethyl)-1-pyrrolidinyl}-2,2,2-trifluoroethanone* [(*S*)**-12**]: To a mixture of (*S*)-**11 **(1.25 g, 6.3 mmol), NEt_3_ (0.77 g, 7.6 mmol) and DMAP (77 mg, 0.63 mmol) in CH_2_Cl_2_ (20 mL), TsCl (1.45 g, 7.6 mmol) was added at 0 °C. The mixture was stirred at room temperature for 3 h. The mixture was diluted with AcOEt (100 mL), and then washed with sat. *aq* NaHCO_3 _(50 mL × 2) and brine (50 mL). The extracts were dried over MgSO_4, _filtered, and concentrated *in vacuo* to give the corresponding tosylate. The crude tosylate was added to a mixture of NaN_3 _(1.03 g, 15.9 mmol) and NaI (190 mg, 1.3 mmol) in DMSO-1,4-dioxane (1:3 v/v, 30 mL) at room temperature. The mixture was stirred at 80 °C for 24 h. After addition of water (50 mL), the mixture was extracted with Et_2_O (50 mL × 3). The organic layers were washed with H_2_O (50 mL × 2) and brine (50 mL). The extracts were dried over MgSO_4, _filtered, and concentrated *in vacuo*. The residue was purified by silica gel column chromatography with hexane/AcOEt (7:1) to afford (*S*)-**12** (1.02 g, 73%). Colorless oil; [α]_D_^26^ -97.7 (*c* 2.3, CHCl_3_); ^1^H-NMR (400 MHz, CDCl_3_) δ 4.26-4.28 (m, 1H), 3.75 (dd, *J* = 12.4, 8.8 Hz, 1H), 3.63-3.72 (m, 2H), 3.48 (dd, 12.4, 2.8 Hz, 1H), 1.94-2.12 (m, 4H) ppm; ^13^C-NMR (126 MHz, CDCl_3_) δ 156.0 (q, *J* = 37.8 Hz), 113.8 (q, *J* = 282 Hz), 58.3, 51.3, 47.5, 27.3, 24.5 ppm; IR (ATR) 2983, 2101, 1685 cm^-1^; MS (FAB) 223(MH^+^, 8), 154 (100); Anal. Calcd. for C_7_H_9_F_3_N_4_O: C, 37.84; H, 4.08; N, 10.52; Found: C, 37.68; H, 4.00; N, 25.44.

*3-Azidobenzamide* (**14b**): A mixture of **13b **(2.62 g, 15 mmol), NH_4_Cl (395 mg, 7.4 mmol), 28% NH_4_OH*aq* (50 mL) and THF (5 mL) was stirred at 50 °C for 24 h. The mixture was extracted with AcOEt (75 mL × 3) and washed with brine (50 mL). The extracts were dried over MgSO_4_, filtered, and concentrated *in vacuo* to give **14b** (1.86 g, 78%). Colorless crystals; Mp 142-143 °C; ^1^H-NMR (400 MHz, CDCl_3_) δ 7.52-7.54 (m, 2H), 7.43 (t, *J* = 8.1 Hz, 1H), 7.18 (ddd, *J* = 8.1, 2.4, 1.0 Hz, 1H) ppm; ^13^C-NMR (126 MHz, CDCl_3_) δ 168.2, 141.0, 135.1, 130.1, 123.4, 122.4, 118.2 ppm; IR (KBr) 3442, 3358, 2111, 1657, 1581, 1443 cm^-1^; MS (FAB) 436 (MH^+^) 163 (100); Anal. Calcd. for C_7_H_6_N_4_O: C, 51.85; H, 3.73; N, 34.55; Found; C, 52.13; H, 3.92; N, 34.53.

*3-(Azidomethyl)benzamide* (**14a**): A procedure similar to that of **14b** afforded **14a** (4.33g, 84%) from **13a**. Colorless crystals; Mp 82-83 °C; ^1^H-NMR (400 MHz, CDCl_3_) δ 7.80 (s, 1H), 7.76 (dt, *J* = 6.4, 2.0 Hz, 1H), 7.46-7.51 (m, 2H), 4.42 (s, 2H) ppm; ^13^C-NMR (126 MHz, CDCl_3_) δ 169.3, 136.1, 134.0, 131.4, 129.1, 127.1, 127.0, 54.2 ppm; IR (KBr) 3369, 3181, 2112, 2086, 1621, 1582 cm^-1^; MS (FAB) 177 (MH^+^, 100); Anal. Calcd. for C_8_H_8_N_4_O: C, 54.45; H, 4.58; N, 31.80; Found; C, 54.43; H, 4.36; N, 32.02.

*(E)-3-Azido-(N-cinnamoyl)benzamide* (**15b**): A mixture of NaH (1.40 g, 59 mmol) and **14b **(3.79 g, 23 mmol) in THF (200 mL) was stirred for 30 min at 0 °C. To a solution of cinnamoyl chloride (3.90 g, 23 mmol) THF (30 mL) was added the resulting mixture, and stirred for 2 h at room temperature. The reaction mixture was quenched with 1 *aq* N HCl (100 mL). The aqueous layer was extracted with AcOEt (150 mL x 3). The combined organic layers were washed with brine (100 mL), then dried over MgSO_4_, filtered, and concentrated *in vacuo*. The residue was purified by recrystallization (CHCl_3_/hexane). The collected mother liquid was purified again by silica gel column chromatography with CHCl_3 _. The desired product **7a **(5.18 g, 76%) was combined. Pale brown crystals; Mp 148-149 °C; ^1^H-NMR (400 MHz, CDCl_3_) δ 8.58 (s, 1H), 7.96 (d, *J* = 15.9 Hz, 1H), 7.83 (d, *J* = 15.9 Hz, 1H), 7.60-7.68 (m, 4H), 7.52 (t, *J* = 7.8 Hz, 1H), 7.43-7.45 (m, 3H), 7.29 (dd, *J* = 2.2, 1.0 Hz, 1H) ppm; ^13^C-NMR (126 MHz, CDCl_3_) δ 167.7, 165.1, 147.1, 141.4, 134.8, 134.5, 130.8, 130.4, 128.9, 128.7, 123.8, 123.6, 119.1, 118.7 ppm; IR (KBr) 3261, 2099, 1703, 1667, 1608, 1350 cm^-1^; MS (FAB) 293 (MH^+^, 100); Anal. Calcd. for C_16_H_12_N_4_O_2_: C, 65.75; H, 4.14; N, 19.17; Found; C; 65.76; H, 4.39; N, 19.13.

*(E)-3-(Azidomethyl)-(N-cinnamoyl)benzamide* (**15a**): A procedure similar to that of **14b** afforded **15a** (6.99 g, 94%) from **14a**. Colorless crystals; Mp 120-121 °C; ^1^H-NMR (400 MHz, CDCl_3_) δ 8.99 (s, 1H), 7.91-7.96 (d, *J* = 15.6 Hz, 1H), 7.85-7.92 (m, 2H), 7.84 (d, *J* = 15.6 Hz, 1H), 7.64-7.67 (m, 2H), 7.53-7.60 (m, 2H), 7.40-7.43 (m, 3H), 4.47 (s, 1H) ppm; ^13^C-NMR (126 MHz, CDCl_3_) δ 168.8, 165.6, 146.8, 136.6, 134.5, 133.6, 132.6, 130.7, 129.4, 128.9, 128.6, 127.7, 127.6, 119.4, 54.2 ppm; IR (CHCl_3_) 3020, 2102, 1681, 1618, 1339, 1216 cm^-1^; MS (FAB) 306 (MH^+^, 97) 131 (100); Anal. Calcd. for C_17_H_14_N_4_O_2_: C, 66.66; H, 4.61; N, 18.29; Found; C; 66.55; H, 4.91; N, 18.11.

### 3.4. General Procedure for Ru-Catalyzed Huisgen Reactions

To a solution of [Cp^*^RuCl]_4_ (2.5 mol%) in DMF, **5** (1.0 eq) and azide (1.0 eq) were successively added at room temperature .The mixture was heated to 110 °C under microwave irradiation with stirring for 20 min. The resulting mixture was diluted with AcOEt and brine, and then extracted with AcOEt twice and washed with brine three times. The extracts were dried over Na_2_SO_4_, filtered, and concentrated *in vacuo*. The residue was purified by silica gel column chromatography.

*tert-Butyl [(1R,2R)-2-[{(1-Benzyl-1H-1,2,3-triazol-5-yl)methyl}(methyl)amino]cyclohexyl] carbamate* (**17a**): White amorphous solid; [α]_D_^28^ -3.1 (*c* 0.95, CHCl_3_); ^1^H-NMR (500 MHz, CDCl_3_) δ 7.57 (s, 1H), 7.29-7.35 (m, 3H), 7.15-7.17 (m, 2H), 5.70 (d, *J* = 15.5 Hz, 2H), 4.60 (br, 1H), 3.52 (d, *J* = 16.0 Hz), 3.47 (d, *J* = 16.0 Hz), 2.20-2.23 (m, 1H), 2.14 (s, 3H), 2.06-2.09 (m, 1H), 1.75-1.79 (m, 2H), 1.65-1.67 (m, 1H), 1.44 (s, 9H), 1.03-1.24 (m, 4H) ppm; ^13^C-NMR (126 MHz, CDCl_3_) δ 155.6, 135.1, 134.7, 134.4, 128.9, 128.2, 127.1, 79.2, 65.5, 51.9, 51.2, 36.2, 33.4, 28.5, 25.1, 24.8, 22.6 ppm; IR (ATR) 3315, 2930, 1701 cm^-1^; MS (FAB) 400 (MH^+^, 80), 344 (100); HRMS (FAB) [C_22_H_34_N_5_O_2_]^+^: 400.2713; Found. 400.2731.

*tert-Butyl [2-[[{1-(2-Hydroxybenzyl)-1H-1,2,3-triazol-5-yl}methyl](methyl)amino]cyclohexyl] carb-amate* (**17b**): Pale brown amorphous solid; [α]_D_^27^ +6.5 (*c* 1.1, CHCl_3_); ^1^H-NMR (400 MHz, DMSO-*d*6) δ 9.81 (s, 1H), δ 8.31 (s, 1H), 7.12 (dd, *J* = 7.6 Hz, 7.6 Hz, 1H), 6.83-6.85 (m, 1H), 6.74 (dd, *J* = 7,6, 7.6 Hz, 2H), 6.40 (br, 1H), 5,59 (s, 2H), 3.75 (d, *J* = 14.4 Hz, 1H), 3.64 (d, *J* = 14.4 Hz, 1H), 3.37 (br, 1H), 2.28-2.33 (m, 1H), 2.08 (s, 3H), 1.59-1.79 (m, 4H), 1.03-1.25 (m, 4H) ppm; IR (ATR) 3387, 3160, 2935, 1661 cm^-1^; MS (FAB) 416 (MH^+^, 100); HRMS (FAB) [C_22_H_34_N_5_O_3_]^+^: 416.2662; Found. 416.2651.

*4-Nitrobenzyl 2-[[5-[[{(1R,2R)-2-(tert-Butylcarbamoyl)cyclohexyl}(methyl)amino]methyl]-1H-1,2,3-triazol-1-yl]methyl]benzoate* (**17c**): Pale brown amorphous solid; [α]_D_^28^ -9.8 (*c* 1.4, CHCl_3_); ^1^H-NMR (400 MHz, CDCl_3_) δ 8.26 (dd, *J* = 8.8, 2.0 Hz, 2H), δ 8.10 (d, *J* = 7.8 Hz, 1H), 7.62 (s, 1H), 7.60 (dd, *J* = 8.8, 2.0 Hz, 2H), 7.50 (dd, *J* = 7.8, 7.6 Hz, 1H), 7.42 (dd, *J* = 7.8, 7.6 Hz, 1H), 6.78 (d, *J* = 7.8 Hz, 1H), 6.12 (d, *J* = 16.6 Hz, 1H), 5,93 (d, *J* = 16.6 Hz, 1H), 5.45 (s, 2H), 4.90 (s, 1H), 3.65 (d, *J* = 14.4 Hz, 1H), 3.47 (d, *J* = 14,4 Hz, 1H), 3.35-3.40 (m, 1H), 2.20-2.23 (m, 2H), 2.14 (s, 3H), 1.71-1.75 (m, 2H), 1.61-1.63 (m, 1H), 1.41 (s, 9H), 1.02-1.27 (m, 4H) ppm; ^13^C-NMR (126 MHz, CDCl_3_) δ 166.1, 155.8, 147.8, 142.8, 137.6, 135.5, 134.1, 133.5, 131.1, 128.54 128.5, 128.2, 127.3, 123.9, 79.0, 66.2, 65.5, 51.5, 49.8, 46.6, 36.2, 33.8, 28.4, 25.1, 24.8, 23.2 ppm; IR (ATR) 2934, 1716, 1523 cm^-1^; MS (FAB) 580 (MH^+^, 100); HRMS (FAB) [C_30_H_39_N_6_O_6_]^+^: 579.2931; Found. 579.2930.

*tert-Butyl [(1R,2R)-2-[Methyl[[1-[{(R)-1-(2,2,2-trifluoroacetyl)pyrrolidin-2-yl}methyl]-1H-1,2,3-triazol-5-yl]methyl]amino]cyclohexyl]carbamate* (**17d**): Pale brown amorphous solid; [α]_D_^26^ -12.3 (*c* 0.92, CHCl_3_);^1^H-NMR (400 MHz, acetone-*d*_6_) δ 7.53 (s, 1H), 5.70 (br, 1H), 4.68 (br, 3H), 3.75 (d, *J* = 14.2 Hz, 1H), 3.73 (d, *J* = 14.2 Hz, 1H), 3.72 (br, 2H), 3.46 (br, 1H), 2.75-2.78 (m, 1H), 2.08 (s, 3H), 1.88-2.00 (m, 2H), 1.77-1.81 (m, 1H), 1.62-1.66 (m, 1H), 1.38 (s, 9H), 1.10-1.38 (m, 4H) ppm; ^13^C-NMR (126 MHz, acetone-*d*_6_) δ 156.7 (q, *J* = 37.2 Hz), 156.3, 136.1, 134.9, 117.3 (q, *J* = 289 Hz), 78.2, 67.6, 59.4, 51.9, 48.2, 48.2, 35.0, 34.7, 28.7, 28.6, 27.2, 26.0, 24.4, 23.6 ppm; IR (ATR) 3370, 2931, 2858, 1683, 1525 cm^-1^; MS (FAB) 489 (MH^+^, 62), 180 (100); HRMS (FAB) [C_22_H_36_F_3_N_6_O_3_]^+^: 489.2801; Found. 489.2802.

*tert-Butyl [(1R,2R)-2-[Methyl[[1-[{(S)-1-(2,2,2-trifluoroacetyl)pyrrolidin-2-yl}methyl]-1H-1,2,3-triazol-5-yl]methyl]amino]cyclohexyl]carbamate* (**17e**): Pale brown amorphous solid; [α]_D_^26^ -17.9 (*c* 1.7, CHCl_3_); ^1^H-NMR (400 MHz, acetone-*d*6) δ 7.53 (s, 1H), 5.57 (br, 1H), 5.03 (br, 1H), 4.52-4.57 (m, 1H), 4.43 (dd, *J* = 13.6, 9.3 Hz, 1H), 3.87 (s, 2 H), 3.75-3.80 (m, 2H), 3.47 (br, 1H), 2.61 (br, 1H), 2.05 (s, 3H), 1.86-2.05 (m, 2H), 1.75-1.80 (m, 1H), 1.65-1.70 (m, 1H), 1.38 (s, 9H), 1.10-1.38 (m, 4H) ppm; ^13^C-NMR (126 MHz, acetone-*d*_6_) δ 156.5 (q, *J_C-F_* = 36.0 Hz), 156.1, 136.4, 134.7, 117.3 (q, *J_C-F_* = 287 Hz), 78.3, 66.4, 59.7, 57.7, 52.0, 48.1, 47.9, 47.5, 35.1, 28.7, 28.7, 27.3, 25.9, 24.4, 23.7 ppm; IR (ATR) 3371, 2933, 2858, 1684, 1522 cm^-1^; MS (FAB) 489 (MH^+^, 100); HRMS (FAB) [C_22_H_36_F_3_N_6_O_3_]^+^: 489.2801; Found. 489.2798.

*tert-Butyl [(1R,2R)-2-[Methyl[2-[1-[{(R)-1-(2,2,2-trifluoroacetyl)pyrrolidin-2-yl}methyl)-1H-1,2,3-triazol-5-yl]ethyl]amino]cyclohexyl]carbamate* (**17f**): Pale brown oil; [α]_D_^26^ -5.5 (*c* 0.77, CHCl_3_); ^1^H- NMR (500 MHz, CDCl_3_) δ 7.54 (s, 1H), 4.95 (br, 1H), 4.59 (d, *J* = 10.9 Hz, 1H), 4.40 (d, *J* = 10.9 Hz, 1H), 4.38-4.40 (m, 1H), 3.66-3.68 (m, 2H), 3.27 (br, 1H), 2.87-2.89 (m, 2H), 2.79-2.81 (m, 1H), 2.62-2.65 (m, 1H), 2.20-2.35 (m, 3H), 2.26 (s, 3H), 1.90-2.02 (m, 2H), 1.62­-1.82 (m, 4H), 1.44 (s, 9H), 1.02-1.25 (m, 4H) ppm; ^13^C-NMR (126 MHz, CDCl_3_) δ 156.4 (q, *J_C-F_* = 37.2 Hz), 155.9, 136.4, 132.6, 116.0 (q, *J_C-F_* = 287 Hz), 78.8, 66.1, 58.8, 52.8, 51.6, 47.3 (q, *J_C-F_* = 3.6 Hz), 47.2, 36.1, 33.4, 28.4, 26.9, 25.3, 24.6, 23.9, 23.3, 22.2 ppm; IR (ATR) 3373, 2932, 1692 cm^-1^; MS (FAB) 503 (MH^+^, 83), 241 (100); HRMS (FAB) [C_23_H_37_F_3_N_6_O_3_]^+^ 503.2879; Found. 503.2882.

*tert-Butyl (1R,2R)-2-[[[1-{3-(Cinnamoylcarbamoyl)benzyl}-1H-1,2,3-triazol-5-yl]methyl](methyl) amino]cyclohexylcarbamate* (**17g**): Pale brown amorphous solid; [α]_D_^26^ +1.4 (*c* 2.0, CHCl_3_); ^1^H-NMR (400 MHz, CDCl_3_) δ 7.85-7.92 (m, 3H), 7.62-7.72 (m 3H), 7.58-7.65 (m, 4H), 7.50-7.53 (m, 3H), 7.40-7.42 (m, 3H), 5.58 (s, 2H), 5.06 (br, 1H), 3.79 (d, *J* = 14.9 Hz, 1H), 3.63 (d, *J* = 14.9 Hz, 1H), 3.30 (br, 1H), 2.25 (br, 2H), 2.17 (s, 3H), 1.91 (m, 1H), 1.78 (m, 1H), 1.66 (m, 1H), 1.36 (s, 9H), 0.90-1.25 (m, 4H) ppm; ^13^C-NMR (126 MHz, CDCl_3_) δ; 167.1 165.6, 155.9, 146.1, 135.9, 134.9, 134.5, 134.2, 133.6, 132.4, 130.5, 129.6, 128.8, 128.4, 128.3, 127.2, 119.6, 79.6, 66.4, 65.7, 51.2, 37.2, 33.7, 28.3, 25.0, 24.7, 22.6, 15.2 ppm; IR (ATR) 3293, 2931, 1679, 1623 cm^-1^; MS (FAB) 573 (MH^+^, 8) 149 (100); HRMS (FAB) [C_33_H_43_N_6_O_4_]^+^: 573.3189; Found. 573.3199

*tert-Butyl (1R,2R)-2-[[2-[1-{3-(Cinnamoylcarbamoyl)benzyl}-1H-1,2,3-triazol-5-yl]ethyl(methyl) amino]cyclohexylcarbamate* (**17i**): Pale brown amorphous solid; [α]_D_^27^ -2.1 (*c* 0.85, CHCl_3_); ^1^H-NMR (400 MHz, CDCl_3_) δ 9.96 (br, 1H), 7.89-7.93 (m 3H), 7.61-7.69 (m, 3H), 7.55 (s, 1H), 7.48 (t, *J* = 7,8 Hz 1H), 7.40-7.42 (m, 4H), 5.69 (d, *J* = 15.6 Hz, 1H), 5.54 (d, *J* = 15.6 Hz, 1H), 4.92 (br, 1H), 2.99 (br, 1H), 2.76 (m, 1H), 2.63 (m, 1H), 2.50 (m, 1H), 2.22 (s, 3H), 2.04-2.22 (m, 3H), 1.60-1.76 (m, 3H), 0.88-1.28 (m, 4H) ppm; ^13^C-NMR (126 MHz, CDCl_3_) δ; 167.1, 165.5, 156.2, 146.4, 135.5, 134.7, 134.0, 133.4, 131.3, 130.6, 129.8, 128.9, 128.6, 128.2, 126.6, 119.5, 67.5, 51.9, 51.8, 51.1, 50.9, 33.6, 28.5, 25.2, 24.7, 23.0 ppm; IR (ATR) 3343, 2927, 2856, 1674 cm^-1^; MS (FAB) 587 (MH^+^, 12) 149 (100); HRMS (FAB) [C_33_H_43_N_6_O_4_]^+^: 586.3346; Found. 586.3340.

### 3.5. General Procedure for Cu-Catalyzed Huisgen Reaction

To a solution of CuSO_4_ (10 mol%) and sodium ascorbate (20 mol%) in *t*-BuOH-H_2_O (1 : 1 v/v), **5a** (1.0 eq) and azide (1.0 eq) were successively added at room temperature. After being stirred for an appropriate time (4-6 h), the mixture was diluted with H_2_O. The residue was extracted with CHCl_3_ three times. The combined organic layers were washed with water twice and brine. The organic phase were dried over Na_2_SO_4_, filtered, and concentrated *in vacuo*. The residue was purified by silica gel column chromatography.

*tert-Butyl [(1R,2R)-2-[[{1-(2-Hydroxybenzyl)-1H-1,2,3-triazol-4-yl}methy](methyl)amino] cyclo- hexyl]carbamate* (**19b**): White amorphous solid; [α]_D_^27^ -2.4 (*c* 1.0, CHCl_3_); ^1^H-NMR (400 MHz, DMSO-*d*_6_) δ 9.82 (s, 1H), 7.15 (dd, *J* = 8.0, 7.6 Hz, 1H), 6.99 (d, *J* = 6.8 Hz, 1H), 6.86 (d, *J* = 8.0 Hz, 1H), 6.76 (dd, *J* = 7.6, 6.8 Hz, 1H), 6.31 (br, 1H), 5,45 (s, 2H), 3.72 (d, *J* = 14.0 Hz, 1H), 3.52 (d, *J* = 14.0 Hz), 3.28 (br, 1H), 2.31-2.36 (m, 1H), 2.11 (s, 3H), 1.86-1.90 (m, 1H), 1.72-1.77 (m, 1H), 1.65-1.70 (m, 1H), 1.36 (s, 9H), 1.11-1.25 (m, 4H) ppm; IR (ATR) 2925, 1715 cm^-1^; MS (FAB) 416 (MH^+^, 100); HRMS (FAB) [C_22_H_34_N_5_O_3_]^+^: 416.2662; Found. 416.2661.

*tert-Butyl [(1R,2R)-2-[Methyl[[1-[{(R)-1-(2,2,2-trifluoroacetyl)pyrrolidin-2-yl}methyl]-1H-1,2,3-triazol-4-yl]methyl]amino]cyclohexyl]carbamate* (**19d**): Pale brown amorphous solid; [α]_D_^24^ -5.6 (*c* 3.3, CHCl_3_); ^1^H-NMR (500 MHz, CDCl_3_) δ 7.43 (s, 1H), 5,11 (br, 1H), 4.70 (dd, *J* = 14.0, 6.3Hz, 1H), 4.59 (dd, *J* = 14.0, 2.9 Hz, 1H), 4.46 (br, 1H), 3.80 (d, *J* = 13.8 Hz, 1H), 3.63 (d, *J* = 13.8 Hz, 1H), 3.40-3.45 (m, 1H)¸ 3.29-3.33 (m, 1H), 2.37-2.39 (m, 1H), 2.28-2.32 (m, 1H), 2.21 (s, 3H), 2.00-2.15 (m, 2H), 1.85-1.90 (m, 2H), 1.78-1.81 (m, 1H), 1.57-1.66 (m, 2H), 1.44 (s, 9H), 1.02-1.31 (m, 4H) ppm; IR (ATR) 3372, 2931, 1692 cm^-1^; MS (FAB) 489 (MH^+^, 100); HRMS (FAB) [C_22_H_36_F_3_N_6_O_3_]^+^: 489.2801; Found. 489.2799.

### 3.6. General Procedure for the Synthesis of Thioureas ***18*** and ***20***

To a stirred mixture of appropriate substrates in CH_2_Cl_2_ at room temperature, TFA was added (CH_2_Cl_2 _: TFA = 1:1). After being stirred at room temperature for 1-3 h, the mixture was made basic with sat. *aq* NaHCO_3 _and extracted three times with CHCl_3_. The combined organic layers were dried over NaSO_4, _filtered, and concentrated *in vacuo* to give the corresponding amine. A solution of the crude amine and 3,5-bis(trifluoromethyl)phenylisothiocyanate (1.0 eq) in THF was stirred at room temperature for 2-10 h. The mixture was concentrated *in vacuo*. The residue was purified by silica gel column chromatography to give the corresponding thiourea. If necessary, the following deprotonation reaction was carried out. To a mixture of the protected compound in THF, LiOH (10 eq) in H_2_O was added (THF/ H_2_O = 1:1). After being stirred at room temperature for 2-10 h, the mixture was quenched with sat. *aq* NaHCO_3_ or sat. *aq* NH_4_Cl. The mixture was extracted three times with AcOEt. The combined organic layers were dried over Na_2_SO_4_, filtered, and concentrated *in vacuo*. The residue was purified by silica gel column chromatography.

*1-[(1R,2R)-2-[{(1-Benzyl-1H-1,2,3-triazol-5-yl)methyl}(methyl)amino]cyclohexyl]-3-{3,5-bis-(tri-*

*fluoromethyl)phenyl}thiourea* (**18a**): White amorphous solid; [α]_D_^27^ +27.1 (*c* 1.5, CHCl_3_); ^1^H-NMR (400 MHz, CDCl_3_) δ 9.00 (br, 1H), 8.08 (s, 2H), 7.60 (br, 1H), 7.52 (s, 1H), 7.42 (s, 1H), 7.21-7.30 (m, 3H), 6.98-7.02 (m, 2H), 5.72 (d, *J* = 15.6 Hz, 1H), 5.59 (d, *J* = 15.6 Hz, 1H), 4.46 (br, 1H), 3.64 (d, *J* = 14.9 Hz, 1H), 3.34 (d, *J* = 14.9 Hz, 1H), 2.37 (s, 3H), 2.27-2.36 (m, 2H), 1.71-1.88 (m, 3H), 1.12-1.38 (m, 4H) ppm; ^13^C-NMR (126 MHz, CDCl_3_) δ 180.4, 141.1, 135.7, 134.02, 133.5, 131.6 (q, *J_C-F_* = 34.8 Hz), 129.1, 128.5, 126.6, 123.1 (q, *J_C-F_* = 274 Hz), 122.3, 117.3, 63.8, 54.1, 52.2, 46.1, 37.8, 33.1, 25.3, 24.9, 22.0 ppm; IR (ATR) 3333, 2935, 1534 cm^-1^; MS (FAB) 571 (MH^+^, 100); HRMS (FAB) [C_26_H_29_F_6_N_6_S]^+^: 571.2079; Found. 571.2075.

*1-{3,5-Bis(trifluoromethyl)phenyl}-3-[(1R,2R)-2-[[{1-(2-hydroxybenzyl)-1H-1,2,3-triazol-4-yl}-*

*methyl](methyl)amino]cyclohexyl]thiourea* (**18b**): White amorphous solid; [α]_D_^24^ +56.0 (*c* 0.56, CHCl_3_); ^1^H-NMR (400 MHz, CDCl_3_) δ 8.27 (br, 1H), 7.60 (s, 2H), 7.52 (s, 1H), 7.49 (s, 1H), 7.26 (s, 1H), 7.23 (br, 1H), 7.15 (dd, *J* = 8.0, 8.0 Hz, 1H), 7.08 (br, 1H), 6.84 (dd, *J* = 8.0, 8.0 Hz, 1H), 6.78 (d, *J* = 8.0 Hz, 1H), 5.58(d, *J* = 14.6 Hz, 1H), 5.28 (d, *J* = 14.6 Hz, 1H), 4.64 (br, 1H), 4.02 (d, *J* = 13.9 Hz, 1H), 3.60 (d, *J* = 13.9 Hz, 1H), 2.63 (m, 1H), 2.40 (m, 1H), 2.18 (s, 3H), 2.02 (m, 1H), 1.91 (m, 1H), 1.88 (m, 1H), 1.77 (m, 1H), 1.74 (m, 1H), 1.14-1.40 (m, 4H) ppm; ^13^C-NMR (126 MHz, CDCl_3_) δ 180.1, 154.1, 139.9, 134.1, 131.9, 131.7, 131.4, 130.6, 123.0, 122.9 (q, *J_C-F_* = 274 Hz), 121.2, 121.0, 118.1, 117.2, 66.5, 54.9, 47.5, 44.3, 38.0, 33.1, 25.0, 24.7, 22.4 ppm; IR (KBr) 3316, 2938, 1540 cm^-1^; MS (FAB) 587 (MH^+^, 100); Anal. Calcd. for C_26_H_28_F_6_N_6_OS: C, 53.24; H, 4.81; N, 14.33; Found: C, 53.14; H, 4.84; N, 14.18.

*2-[[5-[[[(1R,2R)-2-[3-{3,5-Bis(trifluoromethyl)phenyl}thioureido]cyclohexyl](methyl)amino]*

*methyl]-1H-1,2,3-triazol-1-yl]methyl]benzoic Acid* (**18c**): Colorless crystals; [α]_D_^27^ +116 (*c* 0.68, CHCl_3_); Mp 53-54°C; ^1^H-NMR (400 MHz, CD_3_OD) δ 8.17 (s, 2H), 7.85 (d, *J* = 7.6 Hz, 1H), 7.70 (s, 1H), 7.46 (s, 1H), 7.29-7.37 (m, 2H), 7.16 (d, *J* = 7.6 Hz, 1H), 5.93 (d, *J* = 14.4, 1H), 5.74 (d, *J* = 14.4, 1H),4.57-4.61 (m, 1H), 4.24 (d, *J* = 14.2 Hz, 1H), 4.09 (d , *J* = 14.2 Hz, 1H), 1.65-1.72 (m, 2H), 3.16-3.20 (m, 1H), 2.47 (s, 3H), 2.26-2.28 (m, 1H), 2.05-2.09 (m, 1H), 1.83-1.87 (m, 1H), 1.66-1.70 (m, 1H), 1.18-1.46 (m, 4H) ppm; ^13^C-NMR (126 MHz, CDCl_3_) δ 180.9, 173.7, 141.6, 135.4, 134.7, 134.0, 133.3, 132.4, 131.2 (q, *J_C-F_* = 34 Hz), 131.1, 130.9, 129.4, 128.8, 123.3 (q, *J_C-F_* = 277 Hz), 116.8, 68.2, 53.2, 52.1, 38.7, 32.3, 30.4, 29.7, 24.2, 23.0, 22.9 cm^-1^; IR (KBr) 3241, 2942, 1712 cm^-1^; MS (FAB) 615 (MH^+^, 100); HRMS (FAB) [C_27_H_28_F_6_N_6_O_2_S]^+^: 614.1899; Found. 614.1893.

*1-{3,5-Bis(trifluoromethyl)phenyl}-3-[(1R,2R)-2-[methyl[[1-{(R)-pyrrolidin-2-ylmethyl}-1H-1,2,3-*

*triazol-5-yl]methyl]amino]cyclohexyl]thiourea* (**18d**): White amorphous solid; [α]_D_^24^ -0.4 (*c* 0.86, CHCl_3_); ^1^H-NMR (400 MHz, CDCl_3_) δ 8.06 (s, 2H), 7.59 (s, 1H), 7.55 (s, 1H), 4.54 (dd, *J* = 13.7, 6.3 Hz, 1H), 4.35 (dd, *J =* 13.7, 5.9 Hz, 1H), 4.26-4.30 (m, 1H), 3.84-3.89 (m, 1H), 3.71-3.77 (m, 2H), 2.88-3.02 (m, 2H), 2.60 (br, 1H), 2.42-2.47 (m, 1H), 2.23 (s, 3H), 1.71-1.99 (m, 7H), 1.00-1.40 (m, 4H) ppm; ^13^C-NMR (126 MHz, CDCl_3_) δ 180.9, 141.4, 134.4, 131.8 (q, *J_C-F_* = 33.7 Hz), 123.2 (q, *J_C-F_* = 274 Hz), 122.7, 117.3, 64.5, 57.5, 57.4, 54.7, 51.9, 46.1, 36.0, 32.9, 28.8, 25.0, 24.6, 24.5, 21.8 ppm; IR (ATR) 3253, 2935, 2860, 1543 cm^-1^; MS (FAB) 564 (MH^+^, 41), 41 (100); HRMS (FAB) [C_24_H_33_F_6_N_7_S]^+^ 564.2344; Found. 564.2334.

*1-{3,5-Bis(trifluoromethyl)phenyl}-3-[(1R,2R)-2-[methyl[[1-{(S)-pyrrolidin-2-ylmethyl}-1H-1,2,3-*

*triazol-5-yl]methyl]amino]cyclohexyl]thiourea* (**18e**): Pale brown amorphous solid; [α]_D_^27^ +1.0 (*c* 2.6, CHCl_3_); ^1^H-NMR (400 MHz, DMSO-*d*_6_) δ 8.26 (s, 2H), 7.72 (s, 1H), 7.59 (s, 1H), 4.23-4.28 (m, 3H), 3.88 (d, *J* = 14.4 Hz, 1H), 3.66 (d, *J* = 14.4 Hz, 1H), 2.78-2.97 (m, 2H), 2.62-2.68 (m, 1H), 2.12 (s, 3H), 1.16-2.15 (m, 12H) ppm; IR (ATR) 2931, 1692 cm^-1^; MS (FAB) 564 (MH^+^, 100); HRMS (FAB) [C_24_H_33_F_6_N_7_S]^+^: 564.2344; Found. 564.2349.

*1-{3,5-Bis(trifluoromethyl)phenyl}-3-[(1R,2R)-2-[methyl[2-[1-[{(R)-1-(2,2,2-trifluoroacetyl) pyrrol-idin-2-yl}methyl]-1H-1,2,3-triazol-5-yl]ethyl]amino]cyclohexyl]thiourea* (**18f**): Pale brown amorphous solid; [α]_D_^27^ -18.0 (*c* 1.6, CHCl_3_); ^1^H-NMR (500 MHz, pyridine-*d*_5_) δ 11.82 (br, 1H), 8.62 (br, 1H), 8.48 (s, 2H), 7.87 (s, 1H), 7.66 (s, 1H), 4.86 (br, 1H), 4.50 (br, 1H), 4.48 (dd, *J* = 13.8, 4.3 Hz, 1H), 4.62 (dd, *J* = 13.8, 8.3 Hz, 1H), 3.72-3.77 (m, 1H), 2.95-3.02 (m, 2H), 2.44-2.89 (m, 5H), 2.36 (s, 3H), 1.40-1.85 (m, 7H), 0.99-1.35 (m, 4H) ppm; ^13^C-NMR (126 MHz, pyridine-*d*_5_) δ 182.7, 144.4, 138.4, 134.3, 133.2 (q, *J_C-F_* = 32.5 Hz), 125.7 (q, *J_C-F_* = 249 Hz), 118.5, 68.9, 60.6, 57.5, 54.4, 52.9, 48.3, 39.8, 34.6, 31.2, 27.3, 27.2, 26.8, 24.8 ppm (one peak for a nonaromatic carbon is missing); IR (ATR) 3329, 3019, 1735 cm^-1^; MS (FAB) 578 (MH^+^, 50), 369 (100); HRMS (FAB) [C_25_H_34_F_6_N_7_S]^+^ 578.2501; Found. 578.2498.

*3-[[5-[[[(1R,2R)-2-[3-{3,5-Bis(trifluoromethyl)phenyl}thioureido]cyclohexyl](methyl)amino]*

*methyl]-1H-1,2,3-triazol-1-yl]methyl]-N-cinnamoylbenzamide* (**18g**): Colorless crystals; Mp 134-137 °C; [α]_D_^26^ -32.0 (*c* 1.2, CHCl_3_); ^1^H-NMR (500 MHz, acetone-*d*_6_) δ 10.14 (s, 1H), 9.29 (s, 1H), 8.22 (s, 2H), 7.86-7.88 (m, 2H), 7.79 (d, *J* = 15.5 Hz, 1H), 7.58-7.68 (m, 5H), 7.39-7.50 (m, 6H), 5.70 (s, 2H), 4.53 (br, 1H), 3.88 (d, *J* = 14.4 Hz, 1H), 3.74 (d, *J* = 14.4 Hz, 1H), 2.73-2.75 (m, 1H), 2.32-2.35 (m, 1H), 2.25 (s, 3H), 1.96-2.01 (m, 1H), 1.77-1.81 (m, 1H), 1.65-1.69 (m, 1H), 1.18-1.46 (m, 4H) ppm; ^13^C-NMR (126 MHz, CDCl_3_) δ; 180.9, 167.2, 167.0, 145.3, 142,8, 137.8, 135.9, 135.6, 134.9, 134.8, 133.8, 133.2, 131.9, 131.6, 131.3, 129.9, 129.8, 129.1, 128.7, 128.5, 125.4, 123.3, 123.1, 121.5, 117.2, 66.5, 55.6, 51.6, 46.2, 37.8, 33.4, 30.3, 30.1, 25.9, 25.6, 23.8 ppm; IR (ATR) 3127, 1738, 1635, 1528 cm^-1^; MS (FAB) 744 (MH^+^, 31) 369 (100); Anal. Calcd. for C_36_H_35_F_6_N_7_O_2_S: C, 58.13; H, 4.74; N, 13.18; Found: C, 57.73; H, 4.84; N, 12.87.

*3-[[5-[2-[[(1R,2R)-2-[3-{3,5-Bis(trifluoromethyl)phenyl}thioureido]cyclohexyl](methyl)amino]*

*ethyl]-1H-1,2,3-triazol-1-yl]methyl]-N-cinnamoylbenzamide* (**18i**): White amorphous solid; [α]_D_^24^ -161 (*c* 0.74, CHCl_3_); ^1^H-NMR (500 MHz, acetone-*d*_6_) δ 10.15 (s, 1H), 9.22 (s, 1H), 8.27 (s, 2H), 7.91 (d, *J* = 15.9 Hz, 1H), 7.60-7.64 (m, 3H), 7.55 (s, 1H), 7.51 (d, *J* = 15.9 Hz, 1H), 7.39-7.45 (m, 6H), 5.68 (s, 2H), 4.26 (br, 1H), 2.75-2.88 (m, 4H), 2.48-2.52 (m, 1H), 2.30-2.42 (m, 2H), 2.24 (s, 3H), 1.75-2.78 (m, 1H), 1.67-1.70 (m 1H), 1.58-1.61 (m, 1H), 1.18-1.28 (m, 4H) ppm; ^13^C-NMR (126 MHz, CDCl_3_) δ; 180.9, 167.2, 167.1, 145.3, 142.7, 137.9, 136.8, 135.7, 135.0, 133.7, 132.6, 132.1, 131.8, 131.6, 131.3, 130.0, 129.9, 129.1, 128.5, 128.3, 125.5, 123.4, 123.3, 121.5, 117.2, 67.6, 56.2, 52.2, 51.1, 37.6, 33.0, 25.9, 25.5, 23.9, 23.2 ppm; IR (ATR) 3298, 2934, 1726 cm^-1^; MS (FAB) 758 (MH^+^, 30), 369 (100); HRMS (FAB) [C_37_H_38_F_6_N_7_O_2_S]^+^: 758.2712； Found. 758.2717.

*1-{3,5-Bis(trifluoromethyl)phenyl}-3-[(1R,2R)-2-[[{1-(2-hydroxybenzyl)-1H-1,2,3-triazol-4-yl}*

*methyl](methyl)amino]cyclohexyl]thiourea* (**20b**): White amorphous solid; [α]_D_^24^ +39.5 (*c* 0.56, CHCl_3_); ^1^H-NMR (500 MHz, acetone-*d*_6_, 50 °C) δ 8.67 (br, 1H), 8.36 (s, 2H), 7.95 (s, 1H), 7.62 (s, 1H). 7.12 (m, 2H), 6.88 (d, *J* = 7.8 Hz, 1H), 6.76 (t, *J* = 7.5 Hz, 1H), 5.52 (s, 2H), 2.27-2.77 (br, 3H), 2.04 (s, 3H), 1.86-1.95 (m, 2H), 1.68-1.75 (m, 3H), 1.20-1.43 (m, 4H) ppm; IR (KBr) 3266, 3057, 1674 cm^-1^; MS (FAB) 587 (MH^+^, 100); HRMS (FAB) [C_26_H_30_F_6_N_6_OS]^+^ 587.2028; Found. 587.2031.

*1-{3,5-Bis(trifluoromethyl)phenyl}-3-[(1R,2R)-2-[methyl[[1-{(R)-pyrrolidin-2-ylmethyl}-1H-1,2,3-*

*triazol-4-yl]methyl]amino]cyclohexyl]thiourea* (**20d**): Colorless crystals, Mp 179-181 °C; [α]_D_^24^ -0.16 (*c* 3.5, CHCl_3_); ^1^H-NMR (500 MHz, CDCl_3_) δ 8.10 (s, 2H), 7.79 (s, 1H), 7.49 (s, 1H), 4.26 (dd, *J* = 13.8, 5.8 Hz, 1H), 4.19 (dd, *J =* 13.8, 7.5 Hz, 1H), 4.14 (br, 1H), 3.77 (d, *J* = 13.8 Hz, 1H), 3.52 (d, *J* = 13.8 Hz, 1H), 3.36-3.40 (m, 2H), 2.68-2.80 (m, 2H), 2.50-2.57 (m, 1H), 2.32-2.36 (m, 1H), 2.17 (s, 3H), 1.88-1.92 (m, 1H), 1.55-1.77 (m, 5H), 1.05-1.37 (m, 4H) ppm; ^13^C-NMR (126 MHz, CD_3_OD) δ 181.6, 147.4, 143.3, 132.7 (q, *J_C-F_* = 33.6 Hz), 125.3, 124.8 (q, *J_C-F_* = 273 Hz), 123.1, 117.4, 67.2, 67.1, 66.9, 59.4, 56.7, 55.4, 47.1, 37.5, 33.5, 30.1, 26.4, 25.9, 24.2 ppm; IR (ATR) 3375, 2484, 1476 cm^-1^; MS (FAB) 564 (MH^+^, 38), 70 (100); Anal. Calcd. for C_24_H_31_F_6_N_7_S: C, 51.16; H, 5.54; N, 17.24; Found; C, 51.12; H, 5.43; N, 17.24. 

### 3.7. Procedure for Michael addition with Malononitrile

To a solution of malonoritrile (2.0 eq) in CH_2_Cl_2_ (0.2 mL), thiourea-imide **18g** or **18i** (45.5 mg, 60 μmol) in CH_2_Cl_2_ (0.4 mL) was added at room temperature. The mixture was stirred for 48 h. The solution was directly put on the silica gel column without concentration, and purified (hexane/AcOEt = 2:1 to CHCl_3_/MeOH = 10:1) to give a mixture of the product and the starting material. The yield of the product was determined by ^1^H-NMR by a ratio of typical peaks.

### 3.8. General Procudure for Michael addition of Nitrostyrene with Cyclohexanone

To a solution of β-nitrostyrene **22** (0.34 mmol, 1.0 eq) and thiourea **18** or **20** (10 mol%) were added at room temperature cyclohexanone (10 eq), H_2_O (1.0 eq) and AcOH (0.15 eq) successively. The mixture was stirred for 5 h at room temperature. The resulting mixture was directly put on the silica gel column without concentration, and purified by column chromatography. Spectral data of all products **23a-f** were identical with the reported ones [8,38].

## 4. Conclusions

In conclusion, we have described the synthesis of trifunctional thioureas bearing a 1,2,3-triazole tether, in which one of the functional groups is placed at a considerable distance from the thiourea moiety. Regioisomeric catalysts having a 1,5- and 1,4-disubstituted triazole were readily prepared using ruthenium and copper catalyzed Huisgen cycloadditions, respectively. To the best of our knowledge, this is the first reported case of preparation of asymmetric catalysts by Ru-catalyzed azide-alkyne click chemistry [40,41]. We utilized the synthetic thioureas bearing an imide moiety as transition state mimics of the catalytic Michael reaction of α,β-unsaturated imides with malononitrile. Moreover, we demonstrated the catalytic activity of synthesized thiourea-pyrrolidine based catalysts in the enantioselective Michael addition. It was found that thiourea and pyrrolidine functions would synergistically activate substrates, although they are placed at sequentially remote positions (seven atoms’ tether length) to accelerate the reaction rate.
